# Conditional Deletion of *Fgfr3* in Chondrocytes leads to Osteoarthritis-like Defects in Temporomandibular Joint of Adult Mice

**DOI:** 10.1038/srep24039

**Published:** 2016-04-04

**Authors:** Siru Zhou, Yangli Xie, Wei Li, Junlan Huang, Zuqiang Wang, Junzhou Tang, Wei Xu, Xianding Sun, Qiaoyan Tan, Shuo Huang, Fengtao Luo, Meng Xu, Jun Wang, Tingting Wu, Liang chen, Hangang Chen, Nan Su, Xiaolan Du, Yue Shen, Lin Chen

**Affiliations:** 1 Center of Bone Metabolism and Repair, Department of Rehabilitation Medicine, State Key Laboratory of Trauma, Burns and Combined Injury, Trauma Center, Institute of Surgery Research, Daping Hospital, Third Military Medical University, Chongqing 400042, China; 2 Department of Military Nursing, School of Nursing, Third Military Medical University, Chongqing 400042, China; 3 State Key Laboratory of Oral Diseases, West China Hospital of Stomatology, Sichuan University, Chengdu 610041, China

## Abstract

Osteoarthritis (OA) in the temporomandibular joint (TMJ) is a common degenerative disease in adult, which is characterized by progressive destruction of the articular cartilage. To investigate the role of FGFR3 in the homeostasis of TMJ cartilage during adult stage, we generated *Fgfr3*^*f/f*^; *Col2a1-CreER*^*T2*^ (*Fgfr3* cKO) mice, in which *Fgfr3* was deleted in chondrocytes at 2 months of age. OA-like defects were observed in *Fgfr3* cKO TMJ cartilage. Immunohistochemical staining and quantitative real-time PCR analyses revealed a significant increase in expressions of COL10, MMP13 and AMAMTS5. In addition, there was a sharp increase in chondrocyte apoptosis at the *Fgfr3* cKO articular surface, which was accompanied by a down-regulation of lubricin expression. Importantly, the expressions of RUNX2 and Indian hedgehog (IHH) were up-regulated in *Fgfr3* cKO TMJ. Primary *Fgfr3* cKO chondrocytes were treated with IHH signaling inhibitor, which significantly reduced expressions of *Runx2, Col10, Mmp13* and *Adamts5*. Furthermore, the IHH signaling inhibitor partially alleviated OA-like defects in the TMJ of *Fgfr3* cKO mice, including restoration of lubricin expression and improvement of the integrity of the articular surface. In conclusion, our study proposes that FGFR3/IHH signaling pathway plays a critical role in maintaining the homeostasis of TMJ articular cartilage during adult stage.

As a load-bearing and shock-absorbing joint during jaw movement, the temporomandibular joint (TMJ) is frequently used during daily activities in human^1^. Thus, temporomandibular disorders (TMDs) has a high incidence in adult. The most common feature of TMDs is pain that can become chronic and difficult to be cured by conventional approaches. Although TMDs are believed to be linked to the osteoarthritis (OA)-like degenerative changes in mandibular condylar cartilage, the detailed cellular and molecular mechanism of TMJOA remains largely unknown^11^.

TMJ is a specified synovial joint, which is composed of the articular eminence fossa of the temporal bone, the condylar cartilage of the mandible, a fibrocartilaginous disc sandwiched between them, and associated muscles and tendons. The joint space of TMJ is separated by the disk into two parts, the upper and lower articular cavity. During adult stage, although the function of articular cartilage of TMJ is similar to that in other synovial joints in general, the condylar cartilage has unique structure. Mandibular condylar cartilage is characterized as an articular fibrocartilage tissue, structurally divided into four layers including superficial layer, polymorphic layer, flattened chondrocyte layer and hypertrophic layer^2^,^4^. In superficial layer, the antero-posteriorly oriented collagen contributes to the resistance to antero-posterior shear forces^3^. Next, the polymorphic layer containing chondro-progenitors that may serve as a reservoir for the development and maintenance of TMJ cartilage^5^. The deeper layers, including flattened chondrocyte layer and hypertrophic layer, are proteoglycans-enriched cartilage which is suggested to resist compressive forces^6^,^7^,^8^. Therefore, the homeostasis of articular cartilage is critical for the function of TMJ.

OA has been widely studied in limb joints, which is characterized by articular chondrocyte loss, cartilage matrix disequilibrium and subchondral bone loss in the early phase followed by abnormal reparative bone formation producing sclerosis^9^. Studies of TMJ cartilage indicate that the TMJOA has similar pathological processes as those in OA in limb joints, such as hip and knee^6^,^10^. At cellular level, the degraded area of cartilage is increased in condylar cartilage during the early phase of TMJOA^6^,^11^. In addition, the subchondral bone changes also occur, resulting in the abnormal biomechanical property of TMJ that will further exacerbate the deterioration of cartilage homeostasis^12^,^13^. At molecular level, the up-regulated matrix catabolic activity resulting from increased activity of degradative enzymes of the extracellular matrix (ECM), such as matrix metalloproteinases (MMPs) and a distintegrin and metalloproteinase with thrombospondin motifs (ADAMTS), is accompanied by impaired synthesis and abnormal distribution of matrix components, such as collagens and glycosaminoglycans, in articular cartilage during the pathological progression of TMJOA^11^,^14^,^15^.

Fibroblast growth factor receptor 3 (FGFR3) is a critical regulator of skeletal development and growth during embryonic and postnatal stages, especially in cartilage tissues^16^. Patients with activated *FGFR3* mutations have disordered proliferation and differentiation of growth plate chondrocytes, resulting in impaired endochondral ossification at the growth plate leading to skeletal dysplasia, such as achondroplasia (ACH), thanatophoric dysplasia (TD) and hypochondroplasia^17^. Beside the well-studied role of FGFR3 in growth plate chondrocytes, the effect of FGFR3 on the homeostasis of TMJ articular cartilage during adult stage remains largely unclear. In previous study, mice carrying an activated *Fgfr3* (*Fgfr3*^*P244R*^) mutant mimicking Muenke syndrome in human were found to exhibit TMJ cartilage defects^18^. Noteworthily, patients with Muenke syndrome exhibit hypomorphic mandible in which mandibular body length was decreased and gonial angle was increased^19^. The disordered biomechanical properties of TMJ may trigger the dysregulation of MMPs and ADAMTS expression and hypertrophy in articular chondrocytes leading to degenerative changes of the articular cartilage^6^,^12^,^14^. Given that the biomechanical properties of TMJ were already altered during early craniofacial development prior to the postnatal degenerative changes of condylar articular cartilage in *Fgfr3*^*P244R*^ mutant mice, the functional roles and mechanisms of FGFR3 signaling in maintaining articular cartilage of adult TMJ has not been adequately investigated. To determine whether and how FGFR3 signaling affects the maintenance of TMJ articular cartilage homeostasis, we conditionally deleted *Fgfr3* in chondrocytes of mice during adult stage to avoid the involvement of abnormal craniofacial development in the maintenance of TMJ cartilage. These *Fgfr3*-deficient mice exhibit progressive TMJOA-like changes, indicating that FGFR3 signaling is critically involved in the maintenance of the structure integrity and function of TMJ articular cartilage during adult stage.

## Materials and Methods

### Animals

*Fgfr3*^*f/f*^ (*Fgfr3* floxed) mice were previously generated by our group^20^. The *Col2a1-CreER*^*T2*^mice were genotyped as previously described^21^. *Fgfr3*^*f/f*^ mice were crossed with *Fgfr3*^*f/f*^; *Col2a1-CreER*^*T2*^ mice to obtain *Fgfr3*^*f/f*^; *Col2a1-CreER*^*T2*^ (*Fgfr3* cKO) and *Fgfr3*^*f/f*^ (Cre-negative, Control) mice. Tamoxifen (1 mg/10g body weight) was administered by intraperitoneal (i.p.) injection in *Fgfr3* cKO and Cre-negative male littermates for 5 days at 2 months of age^22^. All mice were of the C3H/HeJ background. Animal experiments were performed according to protocols approved by the Laboratory Animal Welfare and Ethics Committee of the Third Military Medical University (Chongqing, China).

### X-ray and micro-CT analysis

X-ray images of TMJ were obtained using an MX-20 Cabinet X-ray system (Faxitron X-Ray, Tucson, AZ, USA). The right side of the undecalcified TMJ specimen was scanned using a vivaCT 40 micro-CT system (Scanco Medical, Brüttisellen, Switzerland). Serial 12.5-μm 2-D and 3-D images were acquired at 70 kV and 113 mA. Constant thresholds (212) were applied to grayscale images to distinguish bone from soft tissue.

### Histological assessment

The left side of TMJ specimen was fixed in 4% paraformaldehyde in 0.1 M phosphate buffer overnight, decalcified in 15% EDTA-phosphate buffered saline at 4 °C and embedded in paraffin. Five μm thick midsagittal sections at 4 different levels (25 μm apart) were cut from the medial compartment of the TMJ, which were stained with Safranin O/Fast Green and hematoxylin and eosin (H&E). These Safranin O/Fast Green- and H&E-stained sections were used to investigate the OA scoring of TMJ articular cartilage using a modified Mankin Score (mRS) system^23^,^24^,^25^,^26^ shown in supplemental information (Table S1). For histomorphometric analysis, the Safranin O-positive cartilage area and thickness were quantified by using Image-Pro Plus 5.1 (Leeds Precision Instruments, Minneapolis, MN, USA). The intervening sections of TMJ were used for other histologic analyses. For tartrate-resistant acid phosphatase (TRAP) staining was performed using a leukocyte acid phosphatase kit (Sigma-Aldrich, St. Louis, MO, USA).

### Immunohistochemical analysis

Decalcified bone sections were deparaffinized with xylene, and endogenous peroxidase activity was quenched by treatment with 3% H_2_O_2_ for 15 min, followed by antigen retrieval by trypsinization for 10 min. Sections were then blocked with normal goat serum for 30 min and incubated at 4 °C overnight with primary antibody followed by the appropriate biotinylated secondary antibody and horseradish peroxidase-conjugated streptavidin-biotin staining. Immunoreactivity was visualized with a 3,3′-diaminobenzidine tetrahydrochloride kit (ZSGB-BIO, Beijing, China) followed by counterstaining with Methyl green. Primary antibodies against the following proteins were used: collagen II (1:400; Chondrex, Redmond, WA, USA), FGFR3 (1:200; Santa Cruz Biotechnology, Dallas, Texas, USA), Osteocalcin (1:200; Santa Cruz Biotechnology), PCNA (1:200; Epitomics, Burlingame, CA, USA), Collagen X (1:200; Abcam, Cambridge, MA, USA), Aggrecan (1:200; Millipore, Billerica, MA, USA), MMP13 (1:200; Proteintech, Chicago, IL, USA), ADANTS5(1:200; Abcam), lubricin (1:600; Abcam), IHH (1:100; Abcam) and RUNX2 (1:200; Santa Cruz Biotechnology). The number of immunoreactive cells in three central regions of condylar cartilage section was counted by using Image-Pro Plus 5.1.

### *In Situ* Hybridization analysis

Decalcified tissues were sectioned at a thickness of 10 μm using a Leica CM3050 S cryostat (Leica Microsystems GmbH, Wetzlar, Germany). Sections were either processed immediately, or stored at −20 °C for later use. *In Situ* hybridization (ISH) was performed using the ISH detection kit (Boster Bio-Engineering Company, Wuhan, China), according to the manufacturer’s instructions. The *Prg4* probe was a 552-bp fragment (nt. 1919–2470) of the mouse cDNA sequence^27^. ISH was visualized with a 3,3′-diaminobenzidine tetrahydrochloride kit (ZSGB-BIO) followed by counterstaining with methyl green.

### Primary chondrocyte cultures

Primary chondrocytes were isolated from the condylar cartilage of 4 week-old mice. Dissected tissues with cartilage were first digested with 0.25% trypsinase (Gibco/Life Technologies, Carlsbad, CA, USA) at 37 °C for 15 min, and then remove muscles, ligaments, and bone tissue. Chondrocytes were isolated from TMJ tissues by additional digestion with 0.1% collagenase II (Gibco/Life Technologies) overnight at 37 °C in a CO_2_ incubator. Cells were seeded in 12-well plates at a density of 2 × 10^5^ cells/well and cultured in Dulbecco’s Modified Eagle’s Medium/F12 (1:1) supplemented with penicillin/streptomycin (Gibco/Life Technologies) and 10% fetal bovine serum until they reached sub-confluence. On day 3 of culturing, primary chondrocytes from both Cre-negative and *Fgfr3* cKO mice were treated with 1 μM 4OH-tamoxifen (Sigma-Aldrich) for 48 h. For IHH signaling inhibitor treatment *in vitro*, the SMOi GDC-0449 (Selleck Chemicals, Houston, TX, USA) was reconstituted in dimethyl sulfoxide (Sigma-Aldrich) and applied in chondrocytes at a final concentration of 1 μM for 24 h after 4OH-tamoxifen treatment, and in the presence of it.

### qRT-PCR

Total RNA was extracted from primary chondrocytes and TMJ cartilage tissues with TRIzol reagent (Invitrogen, Carlsbad, CA, USA) according to the manufacturer’s instructions. All reactions were performed in a Mx3000P thermal cycler (Stratagene, Santa Clara, CA, USA) using the Two-Step QuantiTect SYBR Green RT-PCR Kit (Takara Biotechnology, Otsu, Japan) and reaction conditions were optimized for each gene by altering the annealing temperature (57 °C–61 °C). All samples were measured in triplicate, and each run consisted of samples for genes of interest and cyclophilin A. The forward and reverse primer sequences were as follows: *cyclophilin A*, 5′-CGA GCT CTG AGC ACT GGA GA-3′ and 5′-TGG CGT GTA AAG TCA CCA CC-3′; *Fgfr3*, 5′-CCA CCG ACA AGG AGC TAG AGG-3′ and 5′-CGG TGA CAG GCT TGG CAG TA-3′; *Runx2,* 5′-CCT GAA CTC TGC ACC AAG TC-3′ and 5′- GAG GTG GCA GTG TCA TCA TC-3′; *Col10*, 5′-GCA GCA TTA CGA CCC AAG AT-3′ and 5′-CAT GAT TGC ACT CCC TGA AG-3′; *Mmp13*, 5′-CAG TTG ACA GGC TCC GAG AA-3′ and 5′-CGT GTG CCA GAA GAC CAG AA-3′; *Adamts5*, 5′-GGA GCG AGG CCA TTT ACA AC-3′ and 5′-CGT AGA CAA GGT AGC CCA CTT T-3′; *Gli1*, 5′- GGT CCG GAT GCC CAC GTG AC 3′ and 5′- TCC CGC TTG GGC TCC ACT GT -3′.

### Inhibition of IHH signaling *in vivo*

For *in vivo* experiments, GDC-0449 was reconstituted in 50% (w/v) 2-hydroxypropyl-β-cyclodextrin (Sigma-Aldrich) in water. Mice were injected with tamoxifen (1 mg/10 g body weight) for 5 days at 2 months of age, and were then daily injected with GDC-0449 (1 mg/10 g body weight) or vehicle (50% 2-hydroxypropyl-β-cyclodextrin) intraperitoneally. At 4 months of age, TMJ tissues were dissected for histological analyses.

### Apoptosis assay

The terminal deoxynucleotidyl transferase dUTP nick end labeling (TUNEL) assay was carried out with the *In Situ* Cell Death Detection kit (Roche Applied Science, Pleasanton, CA, USA) according to the manufacturer’s instructions.

### Statistical analysis

Results are expressed as mean ± SD. Differences between groups were analyzed with the Student’s t test. *P* < 0.05 was considered significant.

## Results

### Inducible deletion of *Fgfr3* in TMJ cartilage during adult stage

**Figure 1:**
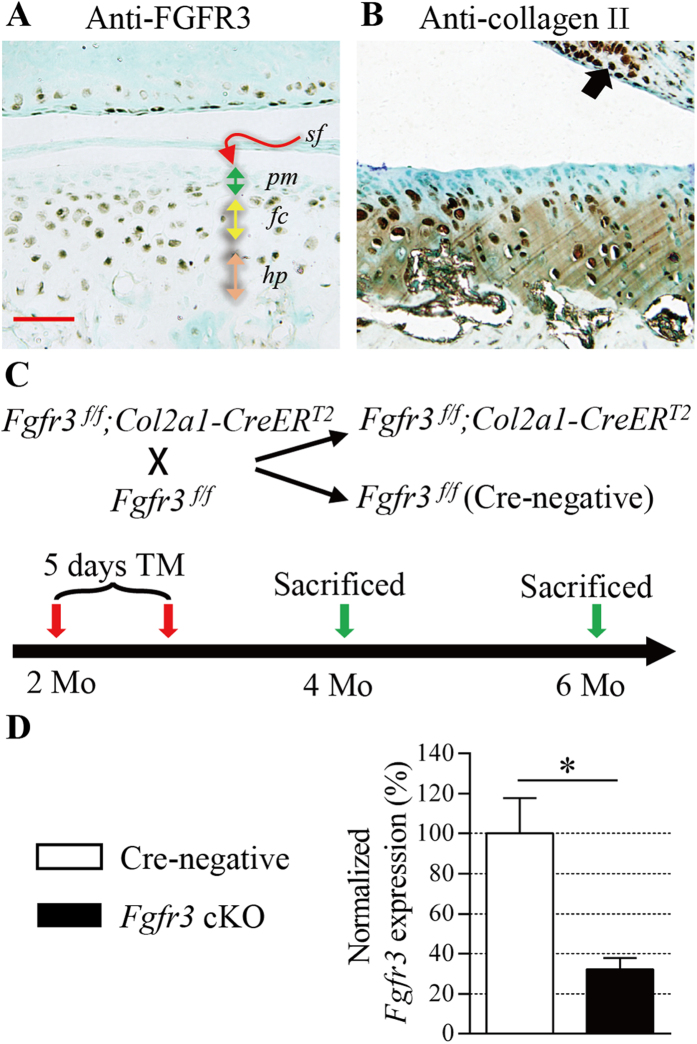
Inducible *Fgfr3* deletion in TMJ cartilage during adult stage. (**A**) FGFR3 IHC was performed in TMJ cartilage of wild-type mice at 2 month of age. Result showing that FGFR3 expression was mainly detected at the flattened chondrocyte layer and hypertrophic layer. (**B**) Collagen II was also mainly expressed at the flattened chondrocyte layer, hypertrophic layer and TMJ disc cells (arrow). (**C**) Scheme of experiment: *Fgfr3*^*f/f*^ mice were crossed with *Col2a1-CreER*^*T2*^ mouse to generate *Fgfr3*^*f/f*^; *Col2a1-CreER*^*T2*^(*Fgfr3* cKO) and Cre-negative control mice. Cre recombination of *Fgfr3* cKO mice was induced by 5-days tamoxifen injection at 2 months of age. TMJ tissues were harvested at 4 and 6 months old. (**D**) *Fgfr3* mRNA expression in TMJ cartilage tissues form *Fgfr3* cKO mice and Cre-negative littermates after tamoxifen administration was investigated by qRT-PCR. Result showing that the efficiency of *Fgfr3* deletion reached 67.63%. Data are expressed as the percent expression relative to controls. Values represent mean ± SD. *p < 0.05 vs. controls (n = 6 TMJs from 3 mice per genotype). *sf:* superficial layer; *pm*: polymorphic layer; *fc*: flattened chondrocyte layer; *hp*: hypertrophic layer. Scale bars = 50 μm.

In adult mice, TMJ condylar cartilage is divided into four layers, which are superficial layer, polymorphic layer, flattened chondrocyte layer and hypertrophic layer along the longitudinal axis (Fig. 1A). Immunohistochemistry (IHC) revealed that the expressions of FGFR3 and collagen II were overlapped at flattened chondrocyte layer and hypertrophic layer (Fig. 1A,B). In previous studies, TMJ chondrocytes were specifically targeted by *Col2a1-CreER*^*T2*^ mice with high efficiency during postnatal stage^28^. To investigate the role of FGFR3 in adult TMJ tissue, *Fgfr3*^*f/f*^ mice were bred with *Col2a1-CreER*^*T2*^mice to generate *Fgfr3*^*f/f*^*; Col2a1-CreER*^*T2*^(*Fgfr3* cKO) and *Fgfr3*^*f/f*^ (Cre-negative, control) mice (Fig. 1C). The efficiency of *Fgfr3* deletion in TMJ condylar cartilage tissues at 2 months of age after 5-days tamoxifen injection, measured by qRT-PCR, reached 67.63% in these *Fgfr3* cKO mice (Fig. 1D). Additionally, the TMJ disc, a specialized fibrocartilaginous tissue, is similar to the meniscus in knee joints. Both of the chondrocyte- and fibroblast-like cell clones was detected in the TMJ disc^29^. The collagen II is also expressed in TMJ disc cells (Fig. 1B), which is in consistent with previous studies^29^,^30^. We found that the expression of FGFR3 in TMJ disc cells was significantly down-regulated in *Fgfr3* cKO mice (Fig. S7A,B,G).

### Conditional deletion of *Fgfr3* in TMJ cartilage leads to OA-like defects

**Figure 2:**
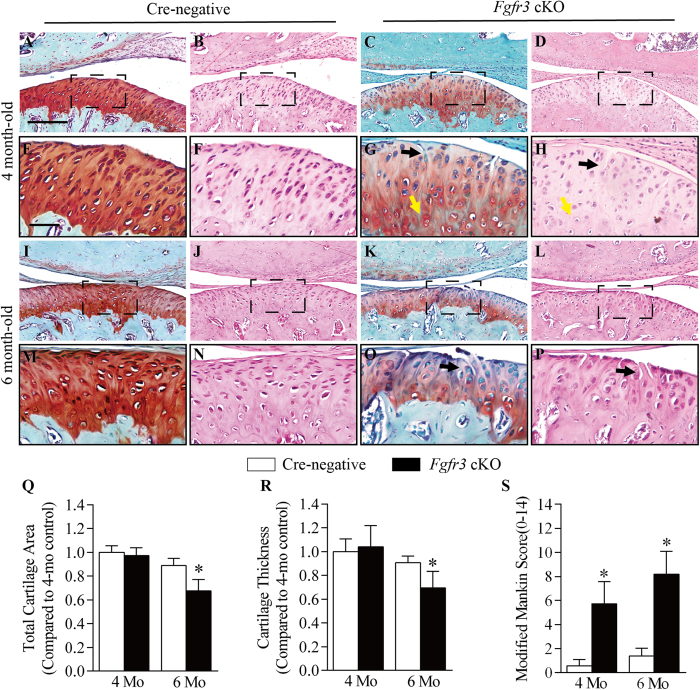
Conditional *Fgfr3* deletion of in TMJ cartilage induces a progressive OA-like defects. TMJ samples were dissected from Cre-negative and *Fgfr3* cKO mice, H&E and Safranin O/fast green staining were performed. (**A–H**) The TMJ cartilage of 4 month-old *Fgfr3* cKO mice presented early features of OA-like defects, including the formation of surface fissures (black arrows) at the superficial layer and polymorphic layer, less Safranin O staining in cartilage, and excessive chondrocyte hypertrophy (yellow arrows) at deeper layers.(**I**–**P**) Severe loss of the cartilage aggrecan, loss volume of the total cartilage and surface fissures (black arrows) were observed in 6 month-old *Fgfr3* cKO mice.(**Q**,**R**,**S**) TMJ cartilage area and thickness were measured by histomorphometry. Analysis of TMJ degenerative changes by a modified Mankin Score (mRS) system. Values represent mean ± SD, *p < 0.05 (n = 6–7 mice per group). Scale bar: 200 μm (**A–D,I–L**); 50 (**E–F,M–P**).

After 5-days tamoxifen injection in *Fgfr3* cKO and Cre-negative mice at 2 months of age, TMJ samples were harvested from these mice at 4 and 6 months of age. No significant gross change was observed in the skull and mandible of *Fgfr3* cKO mice (Fig. S1). Histological analyses revealed that 4 month-old Cre-negative mice exhibited intact structure of TMJ cartilage with evenly stained Safranin O (Fig. 2A,B,E,F). In contrast, 4 month-old *Fgfr3* cKO mice presented early signs of TMJOA, including the formation of surface fissures accompanied by less Safranin O staining at superficial layer and polymorphic layer and the hypertrophy in chondrocytes at the deeper layers (Fig. 2C,D,G,H). At 6 months of age, the cartilage area in the TMJ of *Fgfr3* cKO mice was drastically decreased, and surface fissures were still present at the superficial layer and polymorphic layer with severe loss of Safranin O staining (Fig. 2K,L,O,P). Histomorphometric analysis revealed that the total area and thickness of TMJ cartilage were significantly reduced in 6-month-old *Fgfr3* cKO mice compared to that in Cre-negative mice (Fig. 2Q,R). Furthermore, OA scoring using a modified Mankin Score (mRS) system revealed that the TMJ cartilage degeneration was significantly deteriorated in 4 and 6 month-old *Fgfr3* cKO mice compared to age-matched Cre-negative mice (Fig. 2S). Interestingly, although FGFR3 expression was mainly detected at flattened chondrocyte layer and hypertrophic layer of the TMJ cartilage, the integrity of superficial layer and polymorphic layer was deteriorated in *Fgfr3* cKO mice (Fig. 2G,H,O,P). To determine whether the joint space is involved in these articular surface erosion, X-Ray analysis was performed. No significant change in TMJ joint space was found in 4 and 6 month-old mutant mice (Fig. S2).

Condylar chondrocytes are constantly supplied by chondro-progenitor cells from the superficial layer and the polymorphic layer, and thus are capable of being renewed as that in knee joint cartilage^31^,^32^. In our study, *Fgfr3* gene was only deleted in *Col2a1*-expressed cells at the flattened chondrocyte layer and the hypertrophic layer, chondro-progenitor cells may not be affected in these *Fgfr3* cKO mice. Thus, the degenerated condylar cartilage could be reformed later by wild-type chondro-progenitors from the superficial layer and the polymorphic layer. To address this question, we performed IHC to detect FGFR3 expression in condylar cartilage of *Fgfr3* cKO mice at 2-, 4- and 6-months of age. FGFR3 expression was indeed significantly reduced in *Fgfr3* cKO mice at 2 months old after 5-days tamoxifen injection (Fig. S3A,D,G), while it was significantly up-regulated at 4 and 6 months compared to 2 months in mutant mice (Fig. S3D–F,G). These results indicate that degenerated condylar cartilage was partially renewed by wild-type cells which may be derived from the chondro-progenitor cells. However, the levels of FGFR3 expression in *Fgfr3* cKO mice was still significantly lower than Cre-negative mice at 4 and 6 months old (Fig. S3B,C,E,F,G). Thus, these wild-type chondro-progenitor cells have limited ability in maintaining condylar cartilage.

During OA, the degenerative changes of articular cartilage are considered to be associated with subchondral bone alterations. Tartrate-resistant acid phosphatase and Osteocalcin-IHC staining were performed to assess the number and morphological changes of osteoclasts and osteoblasts at TMJ subchondral bone in Cre-negative and *Fgfr3* cKO mice at 2 months old immediately after TM injection for 5 days. No significant change of osteoclasts and osteoblasts was observed at the subchondral bone of *Fgfr3* cKO mice (Fig. S4). These results suggest that conditional deletion of *Fgfr3* in *Col2a1*-expressed cells did not directly influence osteoblastic bone formation and osteoclastic bone resorption in the subchondral bone. After 2 and 4 months, Micro-CT images of the mandibular condyles of *Fgfr3* cKO mice showed significant subchondral sclerosis, a hallmark sign of OA progression (Fig. S5A). Furthermore, Micro-CT analysis revealed an increased relative bone volume fraction (bone volume/total volume, BV/TV) and trabecular thickness (Tb.Th) at the TMJ subchondral bone of *Fgfr3* cKO mice compared to that of the age-matched Cre-negative mice (Fig. S5B,C). Therefore, these results indicated that subchondral sclerosis may occur secondary to articular cartilage changes in these *Fgfr3* cKO mice.

### *Fgfr3* deficiency impairs TMJ homeostasis

**Figure 3:**
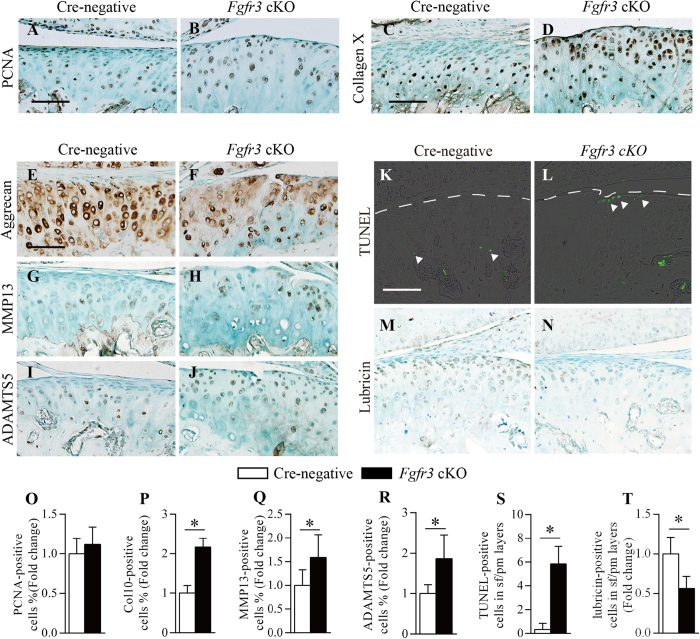
Disturbed homeostasis of TMJ cartilage in *Fgfr3* cKO mice. Sections of TMJ tissue from 4 month-old mice were analyzed. (**A**,**B**) PCNA, (**C**,**D**) Collagen X, (**G**,**H**) MMP13 and (**I**,**H**) ADAMTS5 IHC were performed in TMJ cartilage of Cre-negative and *Fgfr3* cKO mice. (**O**–**R**) The ratios of these immunoreactive positive cells were calculated. Results showing that collagen X, MMP13 and ADAMTS5 expressions were significantly increased, and PCNA expression was no significant change in *Fgfr3* cKO mice compared to Cre-negative mice. Values represent mean ± SD, *p < 0.05 (n = 4 slides per genotype). (**E**,**F**) IHC result showing that aggrecan expression was significantly decreased in *Fgfr3* cKO mice. (**K**, **L**,**S**) TUNEL assay were performed in TMJ cartilage of Cre-negative and *Fgfr3* cKO mice. TUNEL-positive cells (arrowheads) at the superficial layer and polymorphic layer (articular surface, broken line) were counted. Values represent mean ± SD, *p < 0.05 (n = 4 slides per genotype). (**M**,**N**,**T**) The expression of lubricin in TMJ cartilage of Cre-negative and *Fgfr3* cKO mice. The ratio of lubricin-positive cells at the superficial layer and polymorphic layer was calculated. Results showing that lubricin expression was significantly reduced at the articular surface of *Fgfr3* cKO mice. Values represent mean ± SD, *p < 0.05 (n = 4 slides per genotype). Scale bar: 50 μm (**A–N**).

IHC was performed to further investigate the pathological alterations in TMJ cartilage of *Fgfr3* cKO mice. IHC staining for PCNA showed a slightly increased number of positive cells in *Fgfr3* cKO mice compared to that in Cre-negative mice, but did not reach statistical significance (Fig. 3A,O). Furthermore, IHC results revealed an enhanced collagen X expression in TMJ cartilage of *Fgfr3* cKO mice compared to Cre-negative mice (Fig. 3B,P), indicating that loss of FGFR3 induced chondrocyte hypertrophy in TMJ cartilage. IHC results also revealed loss of aggrecan and increased expressions of MMP13 and ADAMTS5, major cartilage matrix-degrading enzymes that lead to cartilage degradation, in TMJ cartilage of *Fgfr3* cKO mice compared to Cre-negative controls (Fig. 3E–J,Q,R). Given the surface of TMJ cartilage in *Fgfr3* cKO mice was remarkably eroded, we performed TUNEL assay to determine whether TMJ chondrocyte apoptosis was increased in the mutant mice. TUNEL-positive cells were mainly distributed at the hypertrophic layer of TMJ cartilage in Cre-negative mice (Fig. 3K). In contrast, there were increased number of TUNEL-positive cells was observed in *Fgfr3* cKO mice, and which is irregularly distributed at the superficial layer and polymorphic layer (Fig. 3L,S). Since lubricin, coded by *proteoglycan 4* (*Prg4*), a critical component of boundary lubrication in the joint fluid, is essentially involved in the protection of TMJ surfaces from degeneration during adult stage^33^,^34^, we performed IHC to detect lubricin expression. In Cre-negative mice, lubricin was abundantly expressed at the superficial layer, polymorphic layer and TMJ discs (Fig. 3M and Fig. S6A), consistent with previous observations^33^,^35^. However, lubricin expression was significantly down-regulated at the articular surface and TMJ discs of *Fgfr3* cKO mice (Fig. 3N,T and Fig. S6B,H). To further confirm the expression pattern of lubricin, *Prg4* mRNA expression was detected by ISH assay. Similar results were observed in *Fgfr3* cKO mice (Fig. S7). Collectively, these results suggest that conditional deletion of FGFR3 in chondrocytes during adult stage induces OA-like degenerative defects in TMJ cartilage due to the changes in cartilage homeostasis.

### Enhanced Indian hedgehog signaling and RUNX2 expression in *Fgfr3* cKO mice

**Figure 4:**
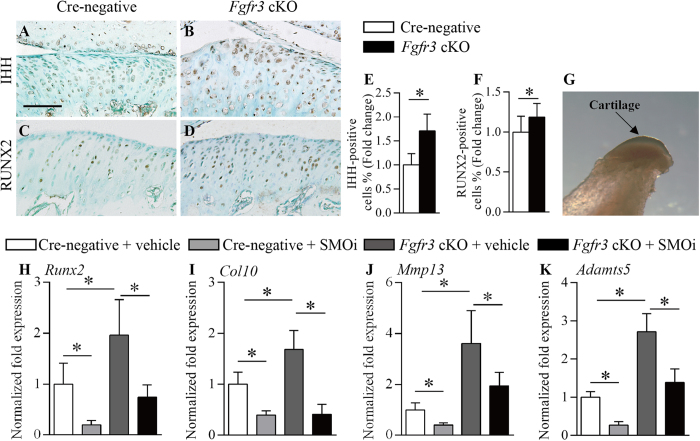
Deletion of *Fgfr3* enhances IHH and RUNX2 expression. Sections of TMJ tissues from 4 month-old mice were analyzed. (**A**,**B**) IHH and (**C**,**D**) RUNX2 IHC were performed in TMJ cartilage of Cre-negative and *Fgfr3* cKO mice. (**E**,**F**) Results showing that the expressions of IHH and RUNX2 were significantly enhanced in TMJ cartilage of *Fgfr3* cKO mice. Values represent mean ± SD, *p < 0.05 (n = 4 slides per genotype). (**G**) Primary chondrocytes were isolated form the translucent cartilage tissues of TMJ (arrow) in 4 week-old mice. (**H**–**K**) qRT-PCR analysis of mRNA expression in Cre-negative control chondrocytes treated with vehicle (dimethyl sulfoxide), Cre-negative chondrocytes treated with IHH signaling inhibitor (1 μM GDC-0449), *Fgfr3*-deficient chondrocytes treated with vehicle and *Fgfr3*-deficient chondrocytes treated with IHH signaling inhibitor. Data are expressed as the normalized fold expression relative to controls. Values represent mean ± SD. *p < 0.05 vs. controls. Scale bar: 50 μm (**A–D**).

Previous investigation implicate that Indian hedgehog (IHH) signaling is involved in OA progression through its regulation of chondrocyte hypertrophy and the expression of cartilage matrix-degrading enzymes^36^. Our IHC analysis revealed an enhanced expression of IHH in TMJ cartilage of *Fgfr3* cKO mice compared to Cre-negative mice (Fig. 4A,B,E). Furthermore, the expression of RUNX2, a critical transcription factor for chondrogenesis, was also up-regulated in mutant mice (Fig. 4C,D,F). To further investigate the role of IHH signaling in TMJOA of *Fgfr3* cKO mice, we used GDC-0449, a selective inhibitor of Smoothened (SMOi)^37^, which can significantly down-regulate the levels of IHH signaling, such as the expression of *Gli1*, in cultured TMJ chondrocytes *in vitro* (Fig. 4G and Fig. S8A) and TMJ tissues *in vivo* (Fig. S8B). Data of RT-PCR revealed that *Gli1*(Fig. S8A), *Runx2* (Fig. 4H), *Col10* (Fig. 4I) *Mmp13* (Fig. 4J) and *Adamts5* (Fig. 4K) expressions were increased by 2.39, 1.96, 1.68, 3.61 and 2.71 folds, respectively, in *Fgfr3* cKO mice compared to Cre-negative mice. After SMOi treatment, the expressions of these genes were significantly reduced in chondrocytes from *Fgfr3* cKO mice (Fig. 4H–K and S8A). Thus, these results indicate that the TMJOA change in *Fgfr3* cKO mice was, at least in part, due to activation of the IHH signaling.

### Ameliorated TMJOA severity in *Fgfr3* cKO mice after SMOi treatment *in vivo*

**Figure 5:**
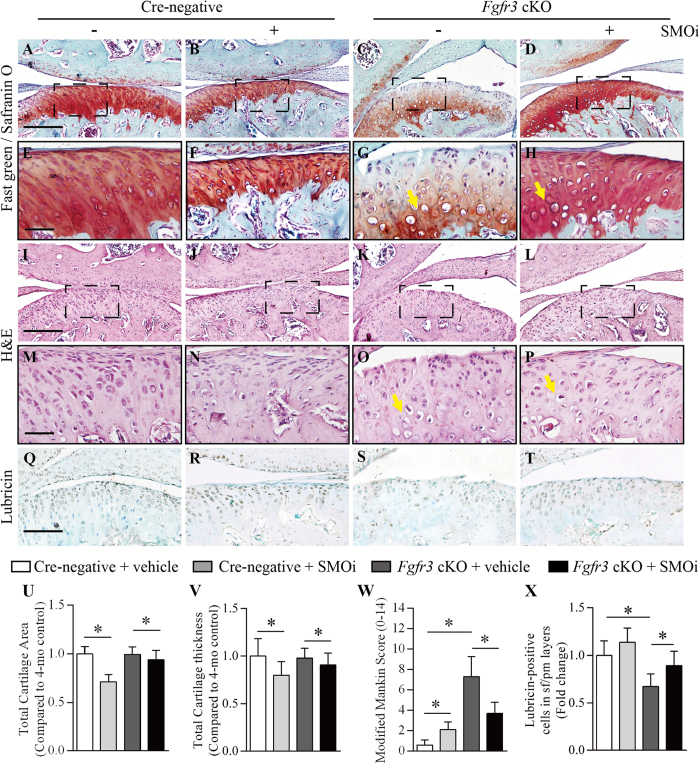
Inhibition of IHH signaling ameliorates TMJOA-like defects in *Fgfr3* cKO mice. (**A**–**H**) Fast Green/Safranin O and (**I–P**) H&E images of TMJ cartilage in Cre-negative mice treated with vehicle (50% w/v 2-hydroxypropyl-β-cyclodextrin) or SMOi and *Fgfr3* cKO mice treated with vehicle or SMOi. Results showing that ameliorated articular integrity and increased aggrecan contents were observed in SMOi-treated *Fgfr3* cKO mice compared to those vehicle-treated mutant mice. Excessive chondrocyte hypertrophy (**G**,**H**,**O**,**P**, yellow arrows) was not completely prevented in TMJ cartilage of *Fgfr3* cKO mice by SMOi treatment. (**U,V**,**W**) The TMJ cartilage area and thickness were measured by histomorphometry. Analysis of TMJ degenerative changes by a modified Mankin Score (mRS) system. Values represent mean ± SD, *p < 0.05 (n = 5–6 mice per group). (**Q**–**T**,**S**) Lubricin IHC was performed in TMJ cartilage of Cre-negative mice treated with vehicle or SMOi and *Fgfr3* cKO mice treated with vehicle or SMOi. Results showing that lubricin expression was significantly increased at the articular surface of *Fgfr3* cKO mice by SMOi treatment. Values represent mean ± SD, *p < 0.05 (n = 4 slides per group). Scale bar: 200 μm (**A–D,M–P**); 100 μm (**Q**–**T**); 50 μm (**E–H,M–P**).

SMOi treatment was performed to investigate whether down-regulation of IHH signaling can alleviate the TMJOA-like defects in *Fgfr3* cKO mice *in vivo*. TMJOA-like phenotypes after SMOi treatment were assessed by histological examination. Histomorphometric results indicated that the articular cartilage area was significantly decreased in Cre-negative and *Fgfr3* cKO mice by SMOi treatment compared to that by vehicle treatment (Fig. 5A–P,U). A similar trend was observed when we analyzed the thickness of TMJ cartilage (Fig. 5A–P,V). Moreover, the mRS system revealed that the severity of TMJOA in *Fgfr3* cKO mice were significantly ameliorated by SMOi treatment (Fig. 5A–P,W). However, excessive chondrocyte hypertrophy was still observed in TMJ cartilage of SMOi-treated *Fgfr3* cKO mice (Fig. 5G,H,O,P). Noteworthily, the formation of surface fissures in *Fgfr3* cKO mice were significantly ameliorated by SMOi treatment (Fig. 5D,H,L,P). Furthermore, IHC analysis revealed that SMOi treatment up-regulated lubricin expression in the articular cartilage and TMJ discs of both Cre-negative and *Fgfr3* cKO mice (Fig. 5Q-T,X and Fig. S6C–F). Above results further support the notion that enhanced IHH signaling is involved in the pathogenesis of TMJOA caused by *Fgfr3* deficiency.

## Discussion

**Figure 6:**
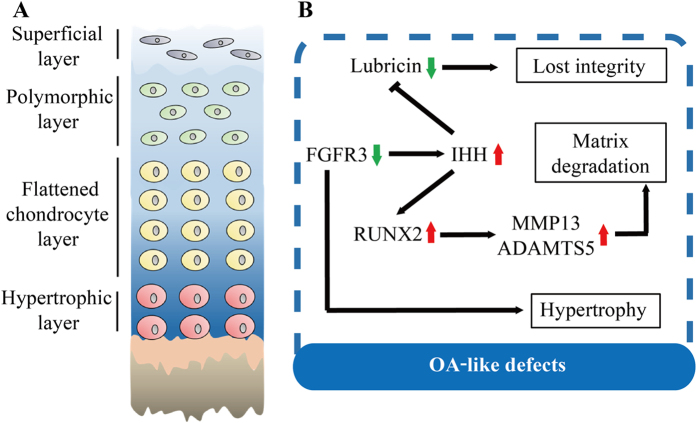
Summary the development of TMJOA-like defects in *Fgfr3* cKO mice. (**A**) Normal structure of the condylar cartilage in TMJ. Mandibular condylar cartilage is well-organized and divided into 4 layers: superficial layer, polymorphic layer, flattened chondrocyte layer and hypertrophic layer. (**B**) FGFR3 is mainly expressed in flattened chondrocyte layer and hypertrophic layer of TMJ cartilage during adult stage. Loss of *Fgfr3* in TMJ cartilage up-regulates IHH expression and promotes chondrocyte hypertrophy. The enhanced IHH signaling down-regulates the expression of lubricin at superficial layer and polymorphic layer leading to disturbance of articular surface integrity, while, up-regulates cartilage matrix-degrading enzymes, such as ADAMTS5 and MMP13, possibly via a RUNX2-mediated manner to degrade the cartilage ECM.

FGFR3 signaling is a critical regulator of cartilage development and maintenance. In previous study, the role of FGFR3 signaling in the development of TMJ articular cartilage was investigated using an activated *Fgfr3* (*Fgfr3*^*P244R*^) mutant mice^18^. However, due to the abnormal craniofacial morphology resulting from the gain-of-function mutation of FGFR3 during skeletal development, the precise role of FGFR3 signaling in the homeostasis of TMJ articular cartilage during adult stage remains elusive. In fact, previous studies have shown that FGFR3 expression is significantly down-regulated in articular cartilage from OA patients compared to that from human without OA, implying that FGFR3 signaling in articular cartilage is involved in the OA process during adult stage^38^. In this study, by using inducible tissue-specific deletion approach, we demonstrated that chondrocyte-specific deletion of *Fgfr3* during adult stage results in TMJOA-like defects, including loss cartilaginous area and accelerated chondrocyte hypertrophy, impaired integrity of articular cartilage surface and progressive subchondral sclerosis. Therefore, our data strongly indicated that FGFR3 signaling is required for the maintaining of TMJ articular cartilage homeostasis during adult stage (Fig. 6).

In previous studies, FGFR3 signaling is considered to exert important anabolic effects on chondrocytes to protect articular cartilage from degeneration^39^,^40^,^41^. However, the mechanism underlying the catabolic effects of FGFR3 signaling on chondrocytes is not fully elucidated. RUNX2, also known as core-binding factor subunit alpha-1, is a critical transcription factor in chondrogenesis, which regulates chondrocyte hypertrophy and the expressions of several ECM-degrading enzymes, such as ADAMTS4-5 and MMP13^36^,^42^,^43^,^44^. Previous study reported that RUNX2 expression is significantly down-regulated in primary chondrocytes of TD patients resulting from a gain-of-function mutation of *FGFR3* compared to normal control^45^. In contrast, we found that RUNX2 expression is up-regulated in TMJ chondrocytes of *Fgfr3* cKO mice, suggesting that RUNX2 may be a downstream molecule of FGFR3 signaling. We thus proposed that the increased RUNX2 expression may be involved in the enhanced expression of MMP13 and ADAMTS5, which may lead to the loss of proteoglycan in TMJ cartilage of *Fgfr3* cKO mice. IHH signaling is also required for the development and homeostasis of TMJ cartilage, as evidenced by the TMJ defects in *Ihh*- and *Gli2*-deficient mice^2^,^35^,^46^. Noteworthily, we have previously reported that there is a decrease in *Ihh* levels in chondrocytes of ACH mice resulting from a gain-of-function mutation of *Fgfr3*, and more importantly, the down-regulation of IHH signaling precedes the appearance of growth plate abnormalities^47^. Moreover, previous results have shown that *Runx2* expression is significantly down-regulated in TMJ chondrocytes of *Ihh*-deficient mice, and Glis, the downstream molecules of IHH signaling, directly up-regulate RUNX2 expression in chondrocytes^35^,^48^. These results imply that FGFR3 may regulate RUNX2 expression via an IHH signaling dependent manner in TMJ chondrocytes. In fact, we found that RUNX2 expression in primary TMJ chondrocytes from *Fgfr3* cKO mice was significantly reduced by IHH signaling inhibitor. Therefore, our data indicate that the enhanced IHH signaling following *Fgfr3* deficiency appears to be responsible for the up-regulated RUNX2 expression in TMJ cartilage tissues.

However, the mechanism for the regulation of IHH expression by FGFR3 signaling remain elusive. Since SHP2, a downstream effector of the receptor tyrosine kinases including FGFR3, activates the ERK MAPK pathway^16^, and recent studies revealed that SHP2 negatively regulates the expression of IHH and the IHH target gene, such as Gli1^49^,^50^,^51^. Furthermore, the similar results are observed by downregulating MAPK signaling^50^,^51^,^52^,^53^. We thus speculate that FGFR3 acts, at least partly, through a SHP2/ERK MAPK-dependent pathway in chondrocytes to regulate the activity of IHH signaling.

Fissure formation at the articular surface was observed in *Fgfr3* cKO TMJ, which is associated with increased chondrocyte apoptosis. Furthermore, the expression of lubricin, a secretory molecule mainly synthesized by chondrocytes at the articular surface and TMJ discs, was significantly down-regulated in *Fgfr3* cKO TMJ. Previous studies reported that mice with loss of lubricin have a normal joint at birth, but develop a progressive OA-like phenotype, including the deterioration of the articular surface in TMJ tissues^33^,^34^. These evidences suggest that lubricin is required for the maintenance of the structure and function of synovial joint during postnatal stage, and imply that the down-regulation of lubricin expression is involved in TMJOA development of *Fgfr3* cKO mice. Interestingly, FGFR3 expression was mainly detected at the flattened chondrocyte layer and hypertrophic layer, but not the articular surface in the TMJ cartilage during adult stage, which is different from the knee joint cartilage where the FGFR3 is expressed in the full thickness^22^,^54^. Therefore, our results indicated that FGFR3 signaling may regulate lubricin expression at the articular surface of TMJ via an indirect manner. Noteworthily, IHH signaling is significantly decreased in TMJ cartilage tissues of mice with gain-of-function mutation of FGFR3 (*Fgfr3*^*P244R*^)^18^. Consistently, we observed that loss of *Fgfr3* in TMJ cartilage led to enhanced IHH protein expression. Moreover, although IHH is restrictively expressed at the flattened chondrocyte layer and hypertrophic layer of normal TMJ cartilage, the receptor of IHH signaling, protein patched homolog (Ptch)1, and the downstream of IHH signaling pathway, such as Gli1, are abundantly expressed at superficial layer, polymorphic layer and TMJ discs^35^. These results indicated that the IHH signaling pathway in chondrocytes of superficial layer and polymorphic layer may be activated by IHH diffused from the underlying flattened chondrocyte layer and hypertrophic layer, which is in consistent with the up-regulated lubricin expression in TMJ cartilage of *Ihh*-deficient mice^35^. We thus speculate that *Fgfr3* deficiency can indirectly impair lubricin expression via enhanced IHH signaling during adult stage, which is involved in the deterioration of TMJ articular surface. However, the previous study showed that HH proteins positively regulate lubricin expression in primary chondrocytes isolated from the superficial zone of knee articular cartilage^55^. Thus, further experiments are needed to investigate the precise mechanism by which IHH signaling affects the lubricin expression at the articular surface.

Given the important role of IHH signaling in the TMJOA development of *Fgfr3* cKO mice, we investigated whether the TMJOA process of mutant mice could be blocked or delayed by inhibiting IHH signaling *in vivo*. Our results showed that TMJOA-like changes in *Fgfr3* cKO mice were ameliorated by SMOi treatment. In previous studies, the inactivation of IHH signaling in chondrocytes reduces the TMJ cartilage area and thickness *in vivo* mainly via the decrease of chondroprogenitors’ mitotic activity^35^. In our studies, the TMJ-deficient phenotypes of *Fgfr3* cKO mice may mainly result from the chondrocyte hypertrophy and the disordered ECM metabolism of the cartilage, which is significantly worse than that were observed in SMOi treatment and *Ihh* cKO mice^35^. In fact, chondrocyte hypertrophy is considered as a key role in the initiation and progression of cartilage degeneration^56^. Thus, we speculate that the hypertrophy and catabolic effects in TMJ cartilage play more critical roles than mitotic activity in TMJOA development. It may explain why the TMJOA-like phenotype of *Fgfr3* cKO mice was partly ameliorated after SMOi treatment, while it decreased cartilage area and thickness in Cre-negative control and mutant mice. Additionally, it is noteworthy that the anti-proliferative effect of IHH signaling inactivation may have less effect on articular chondrocytes of knee joints that have low remodeling activity^57^,^58^. Thus, we suggest that IHH signaling inhibitor play a role as “double-edged sword”, *i.e.*, it inhibits proliferation while reduces ECM catabolism, in TMJ cartilage during adult stage. Further studies are needed to investigate the optimal timing and dosage of IHH signaling inhibitors in order to improve treatment outcome and minimize their detrimental effects on the TMJ cartilage.

Additionally, GDC-0449 was reported to alters intracellular Ca2 + homeostasis in cisplatin-resistant lung cancer cells^59^. Thus, one limitation of our study is that although the well-defined off-target effects of GDC-0449 was not reported in cartilage studies, the non-specific activities are difficult to avoid by using the selective inhibitor compounds of the IHH pathway^60^. In this context, the genetic inactivation of IHH signaling, such as by using *Ihh*^*f/f*^ mice, in *Fgfr3* cKO mice is needed to further support our conclusion in following studies.

In summary, our results indicated that FGFR3 plays an important role in the homeostasis of TMJ cartilage during adult stage, and *Fgfr3* cKO mice is a novel genetic mouse model for the study of TMJOA *in vivo*. Furthermore, we propose that IHH signaling is a critical downstream of FGFR3 signaling to accelerate TMJOA development. Our findings will help to understand the mechanism of TMJOA and to facilitate the development of effective therapies for this common disease.

## Additional Information

**How to cite this article**: Zhou, S. *et al*. Conditional Deletion of *Fgfr3* in Chondrocytes leads to Osteoarthritis-like Defects in Temporomandibular Joint of Adult Mice. *Sci. Rep.* **6**, 24039; doi: 10.1038/srep24039 (2016).

## Supplementary Material

Supplementary Information
