# Supplementary material for: Conditional Deletion of Fgfr3 in Chondrocytes leads to Osteoarthritis-like Defects in Temporomandibular Joint of Adult Mice
Source: Sci Rep. 2016 Apr 4;6:24039. doi: 10.1038/srep24039 (PMC4819201; doi:10.1038/srep24039)
Supplement: Supplementary Information [file srep24039-s1.doc]

**Conditional Deletion of *Fgfr3* in Chondrocytes leads to Osteoarthritis-like Defects in Temporomandibular Joint of Adult Mice**

Siru Zhou^1^, Yangli Xie^1^, Wei Li^2^, Junlan Huang^1^, Zuqiang Wang^1^, Junzhou Tang^1^, Wei Xu^1^, Xianding Sun^1^, Qiaoyan Tan^1^, Shuo Huang^1^, Fengtao Luo^1^, Meng Xu^1^, Jun Wang^1^, Tingting Wu^3^, Liang chen^1^, Hangang Chen^1^, Nan Su^1^, Xiaolan Du^1^, Yue Shen^1^ and Lin Chen^1^

1. Center of Bone Metabolism and Repair, Department of Rehabilitation Medicine, State Key Laboratory of Trauma, Burns and Combined Injury, Trauma Center, Institute of Surgery Research, Daping Hospital, Third Military Medical University, Chongqing 400042, China

2. Department of Military Nursing, School of Nursing, Third Military Medical University, Chongqing 400042, China

3. State Key Laboratory of Oral Diseases, West China Hospital of Stomatology, Sichuan University, Chengdu 610041, China

**Corresponding author's names:**

Professor Lin Chen

1. Center of Bone Metabolism and Repair, Department of Rehabilitation Medicine, State Key Laboratory of Trauma, Burns and Combined Injury, Trauma Center, Institute of Surgery Research, Daping Hospital, Third Military Medical University, Chongqing 400042, China

Tel: 011-86-23-68757041, Fax: 011-86-23-68702991 E-mail: [linchen70@163.com](mailto:linchen70@163.com)

**Table S1**

Modified Mankin Score

| **Feature** | | **Score** |
| --- | --- | --- |
| **Cartilage Erosion Scoring** | | |
| Smooth non-eroded Cartilage  Rough non-eroded Cartilage  Superficial fibrillation  Separation of uncalcified from calcified cartilage  Erosion of uncalcified cartilage only  Erosion extending into calcified cartilage  Erosion down to subchondral bone | | 0  1  2  3  4  5  6 |
| **Pericellular matrix staining (PMS)** | | |
| Normal  Focal points of enhanced intensity  More than 40% of cartilage with enhanced staining | 0  1  2 | |
| **Spatial arrangement of chondrocytes (SAC)** | | |
| Normal  Diffuse hypercellularity  Focal points of hypocellularity (clustering)  Hypocellularity | | 0  1  2  3 |
| **Interterritorial matrix staining (IMS)** | | |
| Normal  Reduced staining  Focal points without any staining  More than 40% of cartilage without any staining | | 0  1  2  3 |


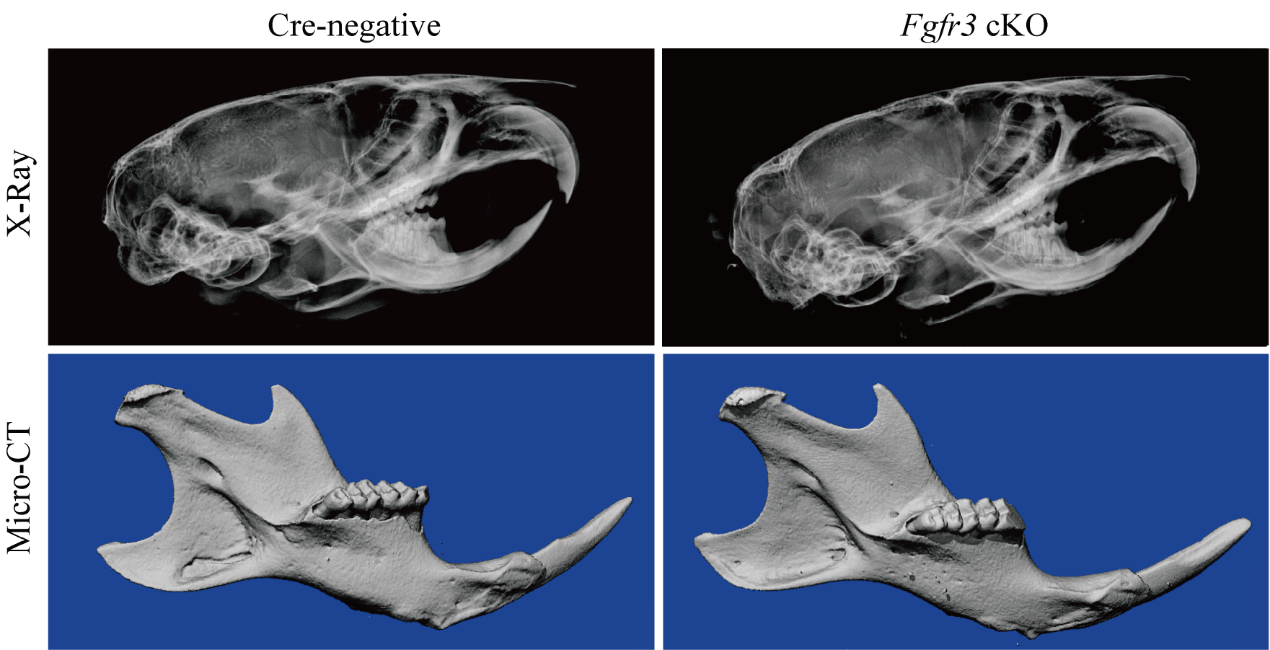


**Figure S1**

**Radiographic assessment of skull and mandible changes in *Fgfr3* cKO mice.** The X-ray and Micro-CT images showing the structure of the skull and mandible in 4 month-old *Fgfr3* cKO mice was not significantly changed.


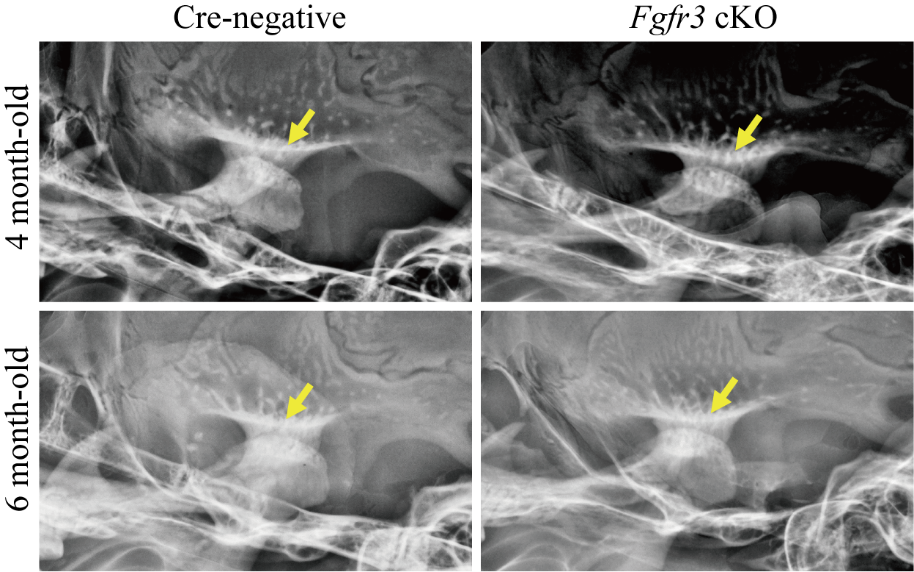


**Figure S2**

**Radiographic assessment of the TMJ space in *Fgfr3* cKO mice.** When the mouth in the closed position, X-ray images showing the TMJ joint space (yellow arrows) was not significantly changed in 4 and 6 month-old *Fgfr3* cKO mice compared to Cre-negative mice.


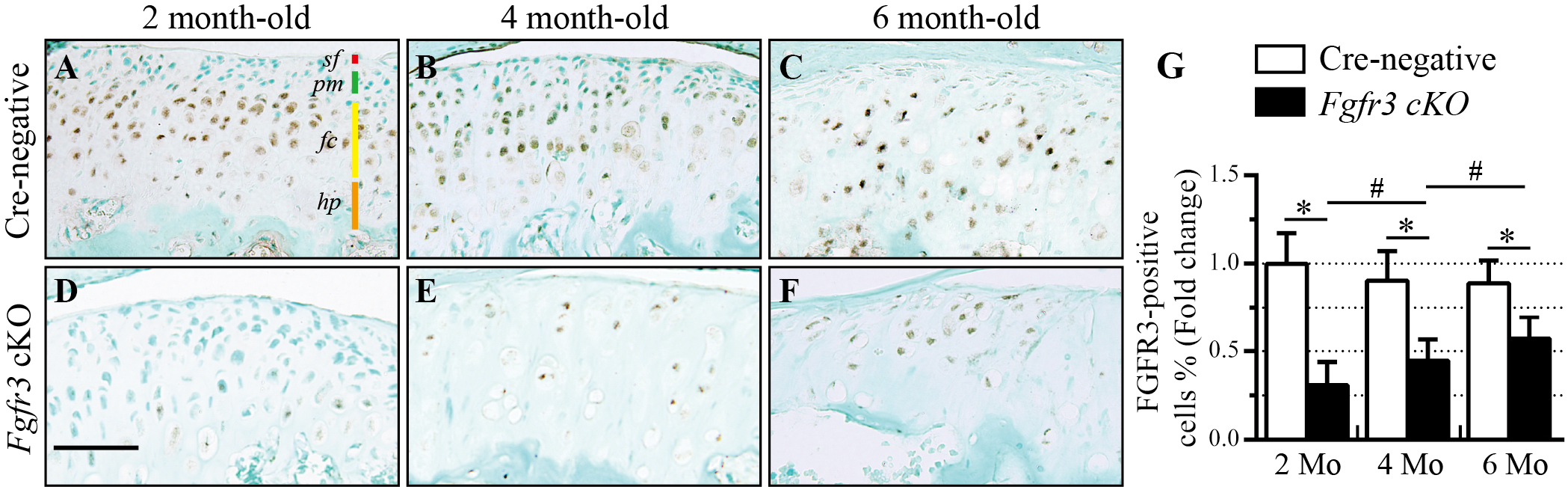


**Figure S3**

**Analysis of FGFR3 expression in *Fgfr3* cKO mice.** FGFR3 immunohistochemical staining were performed at 2, 4 and 6 months old. (**A**-**F** and **G**) Results showing that FGFR3 expression was significantly reduced in *Fgfr3* cKO mice compared to age-matched Cre-negative mice. Values represent mean ± SD, *p < 0.05 (n = 4 slides per genotype). (**D**-**F** and **G**) Results showing that FGFR3 expression was significantly up-regulated in *Fgfr3* cKO mice between 2 and 6 months old. Values represent mean ± SD, #p < 0.05 (n = 4 slides per genotype). *sf*: superficial layer; *pm*: polymorphic layer; *fc*: flattened chondrocyte layer; *hp*: hypertrophic layer. Scale bars = 50 μm.


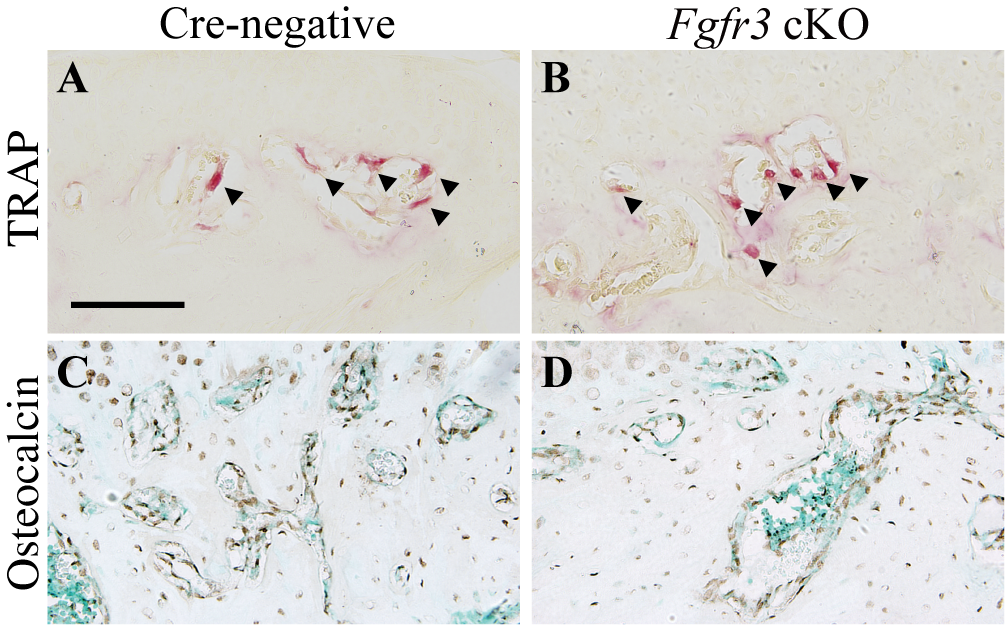


**Figure S4**

**Analysis of osteoclasts and osteoblasts at TMJ subchondral bone in 2 month-old *Fgfr3* cKO mice immediately after 5-days tamoxifen injection.** Tartrate-resistant acid phosphatase (TRAP) and osteocalcin immunohistochemical staining were performed. Osteoclasts (**A** and **B**, arrowheads) and osteoblasts (**C** and **D**) at TMJ subchondral bone were unaffected in *Fgfr3* cKO mice at the early stage of chondrocyte-specific *Fgfr3* deletion. Scale bar: 100 μm (A-D).


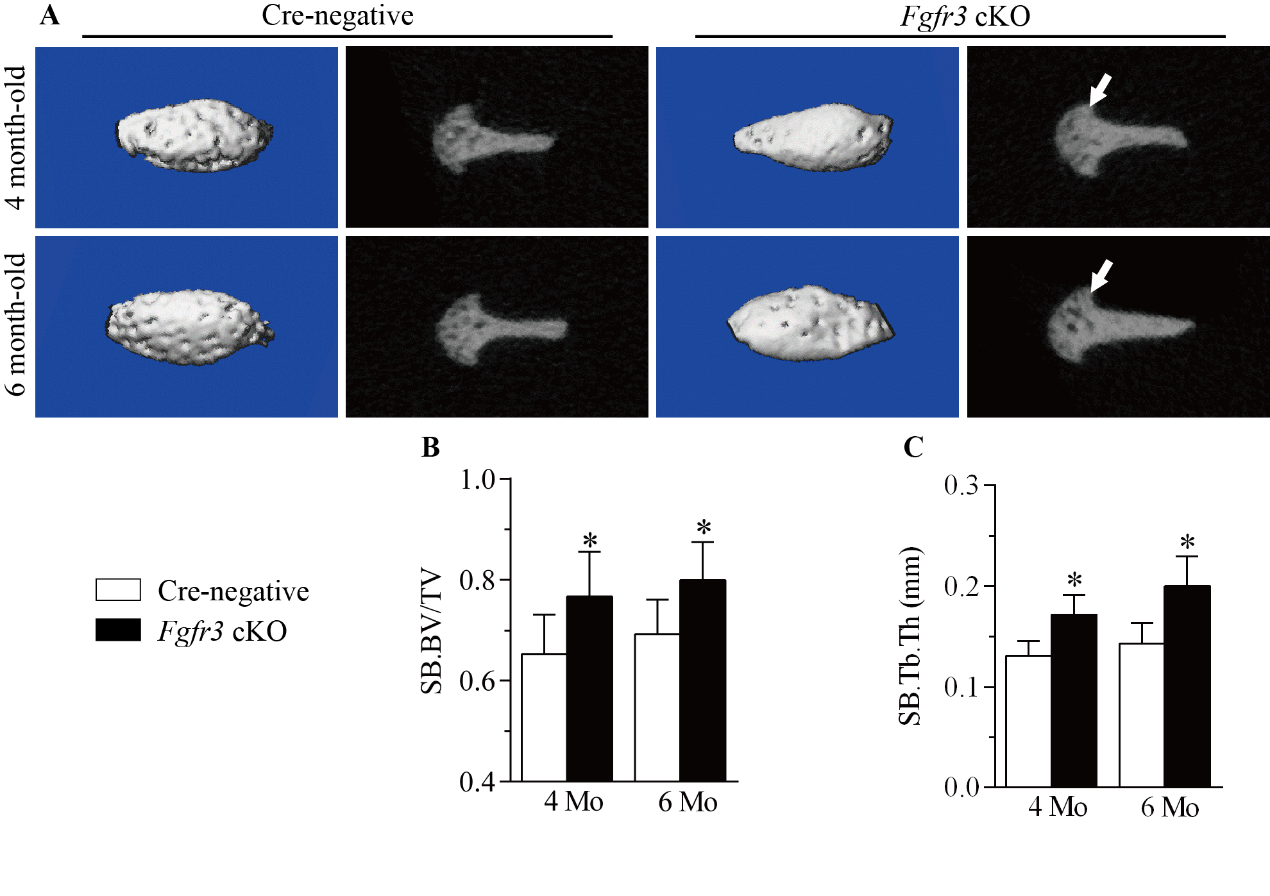


**Figure S5**

**Micro-CT analysis of TMJ subchondral bone in *Fgfr3* deficient mice.** (**A**) Micro-CT images showing that progressive subchondral sclerosis (arrows) was found in 4 and 6 month-old *Fgfr3* cKO mice. (**B**) Quantitative analysis of bone volume/total volume (BV/TV) and trabecular thickness (Tb.Th) in subchondral bone (SB) of Cre-negative and *Fgfr3* cKO mice determined by micro-CT analysis. Values represent mean ± SD, *p < 0.05 (n = 6-7 mice per group).


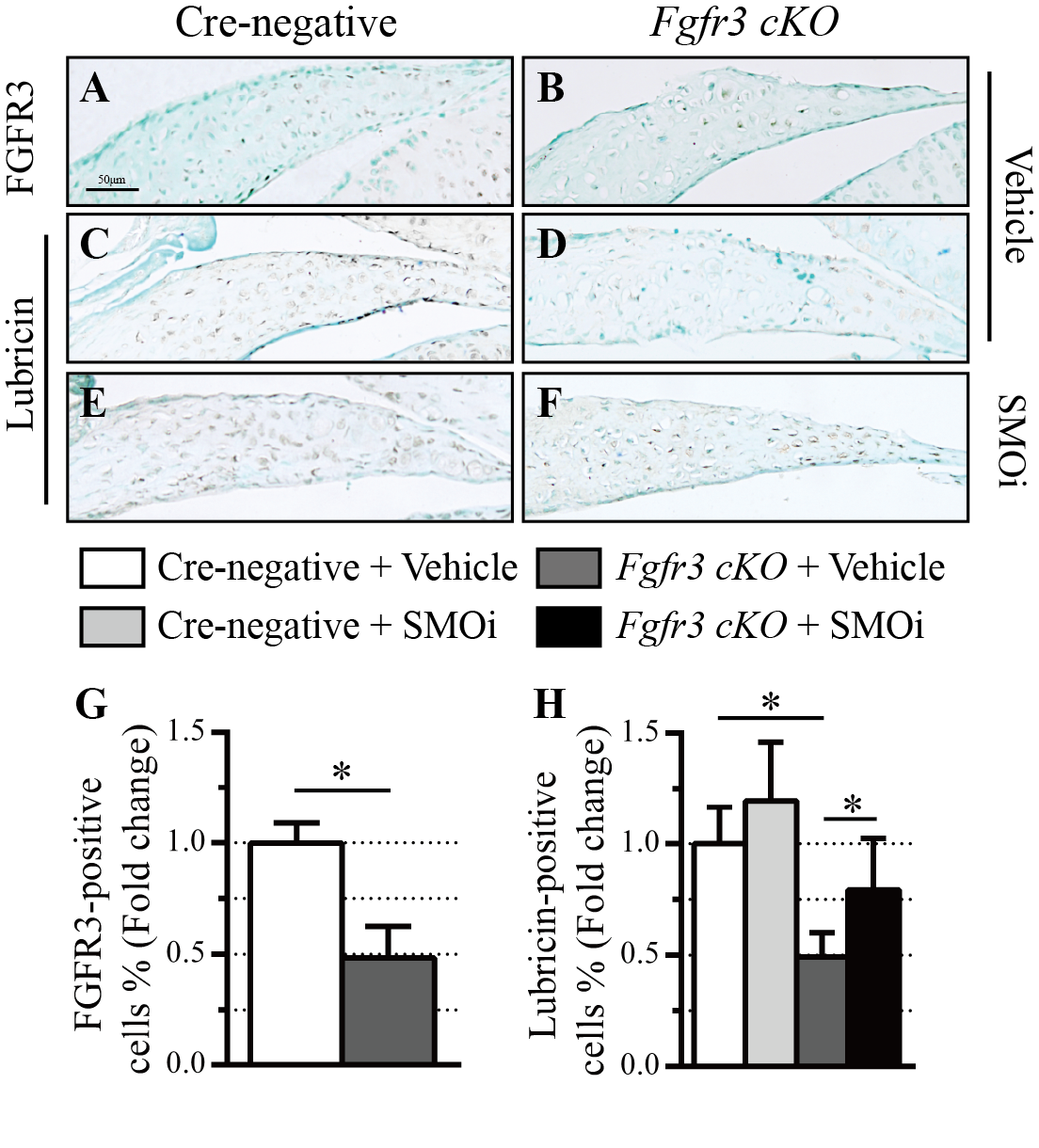


**Figure S6**

**Inhibition of IHH signaling upregulates the levels of lubricin expression in TMJ discs of *Fgfr3* cKO mice.** FGFR3 and lubricin immunohistochemical staining were performed in TMJ discs. (**A**, **B** and **G**) Results showing that FGFR3 expression was significantly reduced in TMJ discs of *Fgfr3* cKO mice compared to that of Cre-negative mice. Values represent mean ± SD, *p < 0.05 (n = 4 slides per genotype). (**C**-**F** and **H**) Results showing that the decreased levels of lubricin expression in TMJ discs of *Fgfr3* cKO mice was significantly upregulated after SMOi treatment. Values represent mean ± SD, *p < 0.05 (n = 4 slides per genotype). Scale bar: 50 μm (A-F).


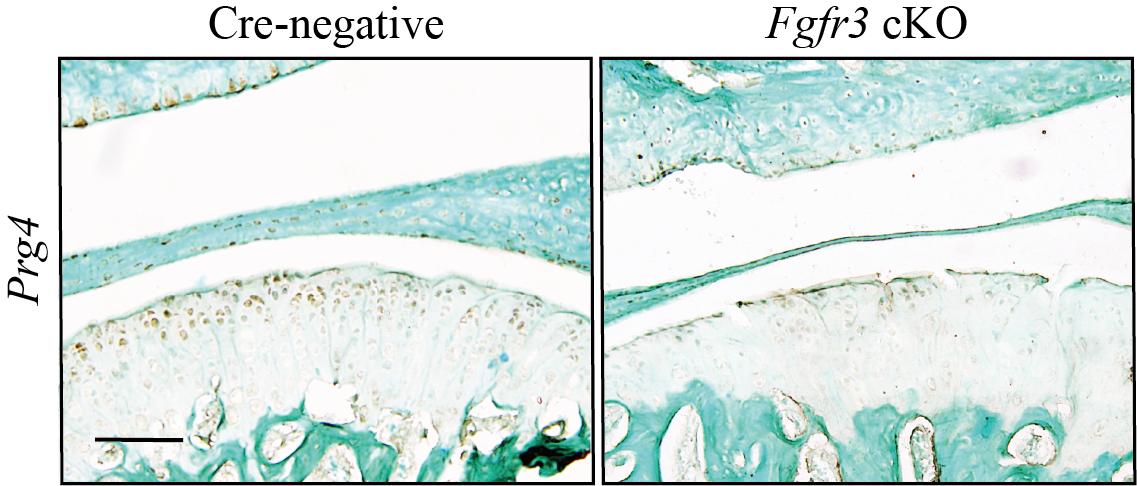


**Figure S7**

**Analysis of *Prg4* mRNA expression in the condylar cartilage at 4 months old.** *In Situ* hybridization assay was performed. *Prg4* mRNA expression was significantly down-regulated in the condylar cartilage of *Fgfr3* cKO mice compared to Cre-negative mice. Scale bar: 100 μm


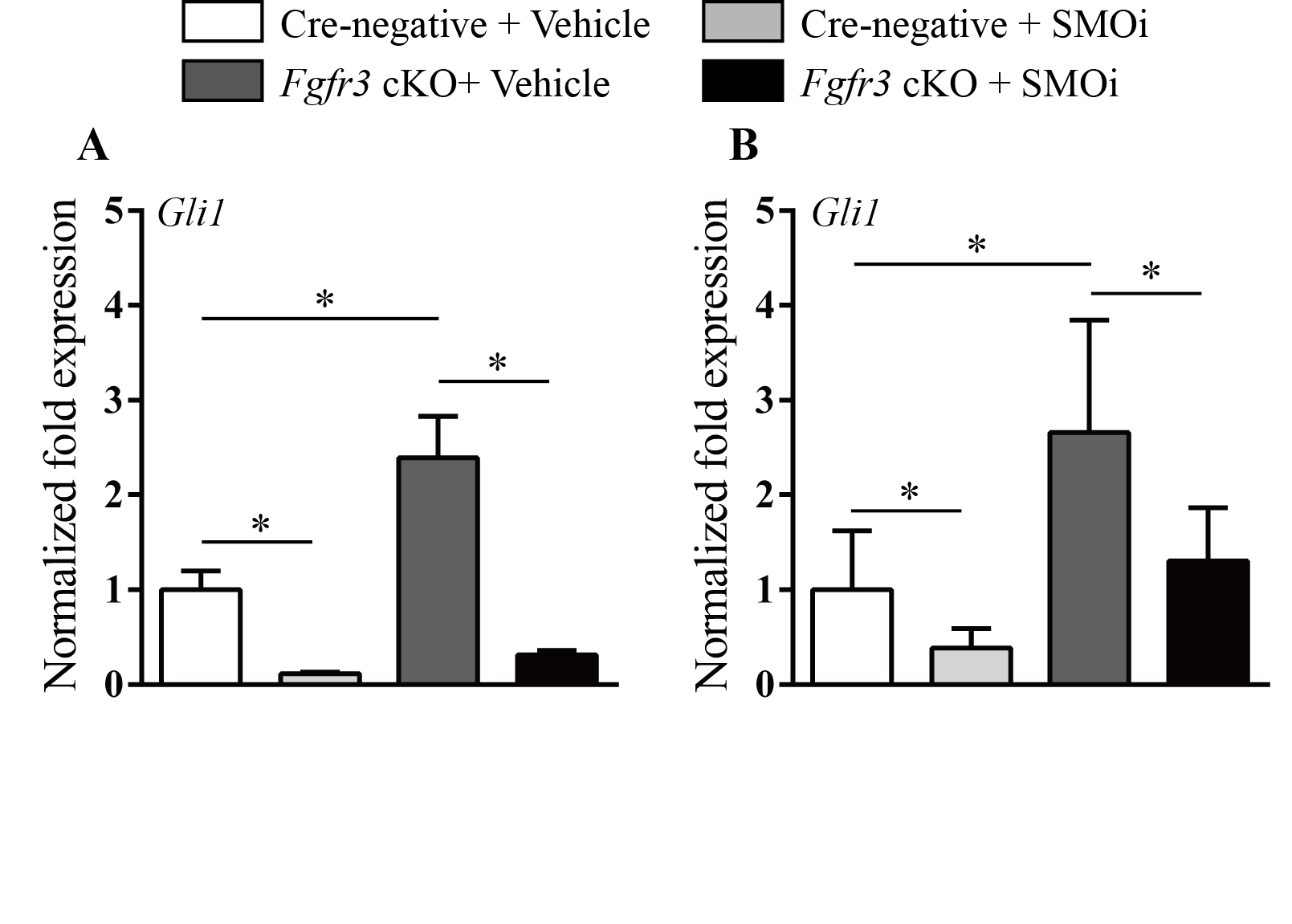


**Figure S8**

**GDC-0449 downregulates the expression of IHH target gene, *Gli1*, in primary chondrocytes and TMJ cartilage tissues from Cre-negative and *Fgfr3* cKO mice.** (**A**) qRT-PCR analysis of *Gli1* expression in Cre-negative control chondrocytes treated with vehicle (dimethyl sulfoxide), Cre-negative chondrocytes treated with 1 μM GDC-0449 (SMOi), *Fgfr3*-deficient chondrocytes treated with vehicle and *Fgfr3*-deficient chondrocytes treated with SMOi. Data are expressed as the normalized fold expression relative to controls. Values represent mean ± SD. *p < 0.05 vs. controls. (**B**) qRT-PCR analysis of *Gli1* expression in TMJ cartilage tissues of Cre-negative mice treated with vehicle (50% w/v 2-hydroxypropyl-β-cyclodextrin), Cre-negative mice after 3-days SMOi treatment, *Fgfr3* cKO mice treated with vehicle and *Fgfr3* cKO mice after 3-days SMOi treatment. Data are expressed as the normalized fold expression relative to controls. Values represent mean ± SD. *p < 0.05 vs. controls (n = 6 TMJs from 3 mice per genotype).
